# Echinoderm Microtubule Associated Protein Like 1 Is Indispensable for Oocyte Spindle Assembly and Meiotic Progression in Mice

**DOI:** 10.3389/fcell.2021.687522

**Published:** 2021-05-28

**Authors:** Hong Yin, Teng Zhang, Hao Wang, Xin Hu, Xuan Hou, Xianbao Fang, Yaoxue Yin, Hui Li, Lanying Shi, You-Qiang Su

**Affiliations:** ^1^State Key Laboratory of Reproductive Medicine, Nanjing Medical University, Nanjing, China; ^2^Shandong Provincial Key Laboratory of Animal Cells and Developmental Biology, School of Life Sciences, Shandong University, Qingdao, China; ^3^Women’s Hospital of Nanjing Medical University, Nanjing Maternity and Child Health Hospital, Nanjing Medical University, Nanjing, China; ^4^Collaborative Innovation Center of Genetics and Development, Fudan University, Shanghai, China

**Keywords:** EML1, oocyte, spindle, meiosis, CDK1, female fertility

## Abstract

Completion of the first meiosis is an essential prerequisite for producing a functionally normal egg for fertilization and embryogenesis, but the precise mechanisms governing oocyte meiotic progression remains largely unclear. Here, we report that echinoderm microtubule associated protein (EMAP) like 1 (EML1), a member of the conserved EMAP family proteins, plays a crucial role in the control of oocyte meiotic progression in the mouse. Female mice carrying an ENU-induced nonsense mutation (c.1956T > A; p.Tyr652^∗^) of *Eml1* are infertile, and the majority of their ovulated oocytes contain abnormal spindles and misaligned chromosomes. In accordance with the mutant oocyte phenotype, we find that EML1 is colocalized with spindle microtubules during the process of normal oocyte meiotic maturation, and knockdown (KD) of EML1 by specific morpholinos in the fully grown oocytes (FGOs) disrupts the integrity of spindles, and delays meiotic progression. Moreover, EML1-KD oocytes fail to progress to metaphase II (MII) stage after extrusion of the first polar body, but enter into interphase and form a pronucleus containing decondensed chromatins. Further analysis shows that EML1-KD impairs the recruitment of γ-tubulin and pericentrin to the spindle poles, as well as the attachment of kinetochores to microtubules and the proper inactivation of spindle assembly checkpoint at metaphase I (MI). The loss of EML1 also compromises the activation of maturation promoting factor around the time of oocyte resumption and completion of the first meiosis, which, when corrected by WEE1/2 inhibitor PD166285, efficiently rescues the phenotype of oocyte delay of meiotic resumption and inability of reaching MII. Through IP- mass spectrometry analysis, we identified that EML1 interacts with nuclear distribution gene C (NUDC), a critical mitotic regulator in somatic cells, and EML1-KD disrupts the specific localization of NUDC at oocyte spindles. Taken together, these data suggest that EML1 regulates acentrosomal spindle formation and the progression of meiosis to MII in mammalian oocytes, which is likely mediated by distinct mechanisms.

## Introduction

Completion of the first meiosis is an essential step toward producing a mature oocyte (commonly known as “egg”) competent for fertilization and supporting preimplantation development. In oocytes of most mammalian species, meiosis stops at diplotene stage around birth, and keeps arrested at this stage until puberty and onward when the surge of preovulatory luteinizing hormone (LH) emerges. Under the stimulation of the LH signaling, meiosis in the fully grown oocyte (FGO) of preovulatory follicles reinitiates, which is manifested by the dissolution of the nuclear envelope and disappearance of the nucleolus, a process also commonly referred to as germinal vesicle breakdown (GVBD). After GVBD, meiosis enters a protracted prometaphase (Pro-MI), during which microtubules nucleate around the condensed chromosomes, and gradually assemble into the bipolar spindles at metaphase I (MI). MI to anaphase I (AI) transition takes place when stable attachment between the kinetochore and microtubule is established, and all homologous chromosomes are aligned at the spindle equator. Homologous chromosome separation starts at AI when the spindle moved to the cortex, and complete segregation is achieved following cytokinesis in telophase I (TI). Meiosis then proceeds to metaphase II (MII) without an intervening interphase in between. In the end, the oocyte first meiosis is completed by formation of a large egg and a small polar body with each of them containing the same number of chromosomes (Eppig et al., 2004; Conti and Franciosi, 2018).

Oocyte meiotic progression is a complex process controlled by sophisticated mechanisms. Central to this process are the correct assembly of bipolar spindles and the establishment of stable attachment between kinetochores and microtubules at MI. These are the key events that ensure the precise timing of meiotic progression and the fidelity of chromosome segregation (Vogt et al., 2008; Mullen et al., 2019). However, in oocytes of most mammalian species, centrosomes are lost during the early stage of oogenesis, and spindles are formed in the absence of centrioles (Szollosi et al., 1972; Manandhar et al., 2005; Schuh and Ellenberg, 2007). The oocyte specialized acentrosomal spindles are assembled with the aid of multiple microtubule organizing centers (MTOCs) containing essential pericentriolar materials (e.g., TUBG/γ-tubulin and PCNT/pericentrin) (Raynaud-Messina and Merdes, 2007; Baumann et al., 2017). This is in contrast to somatic cells and spermatocytes in which spindles are formed under the guidance of centrioles. In addition, unlike in mitotic cells where the sister chromatids are bi-oriented to the opposite spindles poles; in the oocytes, it is the paired homologous chromosomes held together by chiasmata in the bivalents that are bi-oriented at MI. Sister chromatids in the homologous chromosomes are mono-oriented to the same pole in oocytes, which ensures the separation of homologous chromosomes at AI/TI (Holt and Jones, 2009). Therefore, meiotic division in oocytes differs largely from that of spermatocytes and mitotic division in somatic cells, and the oocyte is deemed to use distinct mechanisms to control spindle morphogenesis and chromosome congression. However, these mechanisms remain largely undefined.

Nevertheless, three centrosome-independent pathways are reported to be involved in oocyte microtubule nucleation and spindle assembly: the Ras-like nuclear protein (Ran) GTP-dependent, kinetochore chromosome passenger complex (CPC)-dependent, and Augmin-dependent pathways (Bennabi et al., 2016). The former two pathways mediate the chromosome-based microtubule nucleation and assembly, while the latter pathway drives microtubule amplification through a microtubule-dependent microtubule nucleation mechanism. In the RanGTP pathway, RanGTP produced by the chromosome-bound Ran guanosine exchange factor RCC1 (regulator of chromosome condensation 1) forms a gradient around the chromosome. This RanGTP gradient promotes the release of Importin-bound spindle assembly factors, such as TPX2 and NuMA (Brunet et al., 2008; Kolano et al., 2012), and allow them to regulate oocyte spindle morphogenesis. Although the RanGTP pathway is initially found to be indispensable for only human oocyte spindle assembly (Holubcova et al., 2015), a recent study demonstrates that it is also crucial for mouse oocyte spindle formation (Drutovic et al., 2020). The CPC is composed of the Aurora B/C, INCENP (inner centromeric protein), Survin, and Borealin, which are localized at the kinetochore and are all crucial for the formation of normal bipolar spindles and meiotic progression in oocytes (Sharif et al., 2010; Sun et al., 2010; Jiang et al., 2014; Nguyen et al., 2018). Augmin is a hetero-octameric protein complex comprised of HAUS 1–8, which triggers new microtubule growth by recruiting γ-tubulin to pre-existing microtubules. Augmin is reported to be crucial for microtubule nucleation and bipolar spindle formation in *Xenopus* egg extracts and *Drosophila* oocytes (Petry et al., 2011; Colombie et al., 2013), but its function in mammalian oocytes remains largely unclear. Given the enormous size difference between somatic cells and oocytes (∼40 μm in diameter of Hela cells vs. 85 and 120 μm in diameter of mouse and human oocytes), these spatial restricted microtubule nucleation and assembly pathways are critical for the oocyte to manage the progression of meiosis within such a large volume. It is therefore not surprising that the oocyte adopt a meiosis-specific mechanism to regulate these pathways. Indeed, a conserved liquid-like meiotic spindle domain (LISD) is identified recently in mammalian oocytes that specifically regulate meiotic spindle assembly (So et al., 2019). This oocyte-specific LISD is formed by multiple microtubule regulatory factors (e.g., microtubule nucleation and stability regulator TACC3 and CHC17) through phase separation, disruption of which causes severe spindle assembly defects. Formation of such a LISD may be of particular advantage to the oocyte with such a large volume to enrich microtubule regulatory factors in local proximity to the spindle.

Fine-tuned interactions between factors that regulate microtubule dynamics and function are essential for oocyte spindle morphogenesis and chromosome segregation (Greaney et al., 2018; So et al., 2019). As a major type of microtubule regulatory factors, microtubule-associated proteins (MAPs) are frequently found to be expressed in oocytes and considered to play a crucial role in the control of oocyte acentriolar spindle assembly and meiotic progression (Han et al., 2010; Goodson and Jonasson, 2018). Interestingly, an ever-growing number of MAPs has been identified in oocytes of several model organisms (Gache et al., 2010; Han et al., 2010). However, the identity of the MAPs that are critical for oocyte meiotic maturation and the extent to which these essential MAPs contribute to the regulation of oocyte spindle morphogenesis remain largely unclear. Nevertheless, a recent study in our laboratories indicated that the echinoderm microtubule associated protein (EMAP) like (EML) family proteins are probably such essential MAPs that are crucial for oocyte meiotic spindle formation and meiotic progression in the mouse (Yin et al., 2020).

EMLs are a highly conserved family of MAPs, with 6 members (*i.e.*, EML1-6) found in mammals (Suprenant et al., 2000). They are considered to be a unique and important class of MAPs owing to lower sequence homology with other commonly known MAPs. The founding member of this family protein, *i.e.*, EMAP, was initially discovered as the principal components of the microtubule cytoskeleton in sea urchin eggs and embryos in the early 1990s (Suprenant et al., 1993; Hamill et al., 1998). Since then, for quite a long time, the only common features recognized for this family of proteins had remained to be decorating spindles and regulating microtubule stability *in vitro* (Suprenant et al., 1993; Eichenmuller et al., 2002; Tegha-Dunghu et al., 2008; Kielar et al., 2014; Chen et al., 2015; Richards et al., 2015). Until recently, the physiological role of this family protein was starting to be appreciated, as the disease- and developmental disorder causing- mutations and deletions of the EML encoding genes were gradually discovered (De Keersmaecker et al., 2005; Lin et al., 2009; Sun et al., 2015). Of particularly, for instance, deletions or losses of function of EML1 in mouse, rat, and human cause subcortical heterotopia in the brain or disorganization of retina architecture in the eye, which is probably brought by defects in primary cilia formation, mitotic spindle length/positioning and proliferation of neuronal progenitors, and lamination of the inner retina (Eudy et al., 1997; Kielar et al., 2014; Bizzotto et al., 2017; Collins et al., 2019; Oegema et al., 2019; Uzquiano et al., 2019; Collin et al., 2020; Grosenbaugh et al., 2020). Unfortunately, these interesting new findings were all on somatic cells, the function of EMLs in oocytes remained virtually unknown. Nevertheless, we found recently that all the six members of EML family were expressed in the ovarian follicles although their abundance varies in the oocyte and granulosa cells. Knockdown of EML6, the mostly preferentially expressed EML protein by oocytes, impaired spindle integrity and the fidelity of chromosome segregation (Yin et al., 2020). Therefore, EMLs are crucial for the control of oocyte spindle morphogenesis and meiotic division. Here, as a continuation of our recent effort to unraveling the role of EMLs in mammalian oogenesis, we show that EML1, another member of the EML family, is also essential for oocyte maturation and female fertility by regulating oocyte spindle assembly and the progression of meiosis to MII via distinct mechanisms.

## Materials and Methods

### Ethics Statement

All animal procedures and experiments were approved by the Ethical Committee of Laboratory Animals and the Animal Care and Use Committee of Nanjing Medical University (NJMU), and were carried out in accordance with the institutional guidelines of Animal Care and Use.

### Animals

The ENU-induced *Eml1*^tvrm360^ point mutant mice were provided by Dr. Patsy Nishina at The Jackson Laboratory. These mice were imported to the investigator’s colony at NJMU, and were maintained on identical C57BL/6J genetic background. Mice were genotyped by PCR using primers P1 (CCCATGACAACTGCATCTACATATGA), P2 (GCAATCGGCTGGCATGACAACTGCATCTACATGTAT) and P3 (GGTAAGTTTCTCTTGCCTTTCTGA), with P1 + P3 amplifying a 168-bp product specific to the mutant allele, and P2 + P3 amplifying a 178-bp wildtype allele. Normal wildtype ICR mice were purchased from the Animal Core Facility of Nanjing Medical University, while the C57BL/6JXDBA2 (B6D2) F1 mice were produced at the investigator’s own colony.

### Chemicals and Reagents

Unless otherwise specified, all chemicals and reagents were purchased from Sigma-Aldrich (United States). Antibodies used in this study include: rabbit polyclonal anti-EML1 (1:100, Proteintech, #12765-AP), rabbit polyclonal anti-EML4 (1:500, Cell Signaling Technology, #2428), rabbit polyclonal anti-Gamma Tubulin (1:200, Proteintech, #15176-AP), rabbit polyclonal anti-Pericentrin (1:200, Abcam, # ab4448), rabbit polyclonal anti-Phospho-cdc2 (Tyr15) (1:500, CST, #9111); rabbit monoclonal anti-BubR1(1:250, Abcam, #ab3305), rabbit polyclonal anti-tRFP/mKate (1:500, evrogen, #AB234), mouse monoclonal anti-Cyclin B1 (1:100, abcam, #ab72), mouse monoclonal anti-Cdk1/Cdk2 (1:100, Santa Cruz, #sc-53219), mouse monoclonal anti-NUDC (1:100, Santa Cruz, #sc-365782), human anti-Centromere (1:500, Antibodies Incorporated, #15-234), and Alexa flour 594/488-conjugated secondary antibodies (Thermo Fisher Scientific, United States). Rhodamine Phalloidin (1:750, #R415) and PD166285 (#3785) were purchased from Thermo Fisher Scientific and Tocris Bioscience, respectively.

### Fertility Test

To assess the reproductive potential, 8-week-old wide type (WT) control (*n* = 3) and *Eml1*^tvrm360^ mutant (*n* = 3) female mice were mated with normal adult B6D2F1 males at the ratio of 1:1. Although most of the *Eml1*^tvrm360^ mutant mice died at about 4 months of age, mating pairs were continuously caged together for a period of 2 months. The number of pups for each litter was recorded at birth, and the average accumulating number of pups per female was calculated at the end of the fertility test.

### Oocyte Isolation and Culture

Fully grown oocytes (FGOs) were isolated from large antral follicles of 22-days old female mice that were initially primed with equine chorionic gonadotropin (eCG, Ningbo Second Hormone Factory) for 46 h, and were matured in culture as described previously (Yin et al., 2020). The medium used for oocyte collection and culture was bicarbonate-buffered minimum essential medium with Earle’s salts (Thermo Fisher Scientific) supplemented with 75 μg/ml penicillin G, 50 μg/ml streptomycin sulfate, 0.23 mM pyruvate, and 3 mg/ml bovine serum albumin (BSA). Oocyte culture was carried out at 37°C in an Eppendorf NewBrunswick Galaxy170R incubator (Hamburg) infused with 5% O_2_, 5% CO_2_ and 90% N_2_. During the culture, resumption and completion of the first meiosis were assessed by scoring the oocytes that have undergone germinal vesicle breakdown (GVB) and first polar body (PB1) extrusion, respectively. To study the subcellular localization of EML1 in oocytes during meiotic maturation, oocytes were collected at the individual timepoint of 0, 4, 6, 8, 10, and 14 h that corresponds to the meiotic stages of GV, Pro Metaphase I (Pro-M I), M I, Anaphase I (A I), Telophase I (T I), and Metaphase II (M II), respectively, and processed for immunofluorescence (IF) analysis. For Western Blot analysis of EML1 at GV, M I and M II stages, oocyte samples were collected at 0, 6 and 14 h respectively.

To examine the meiotic status of the ovulated oocytes, 7 weeks old WT and *Eml1*^tvrm360^ mutant female mice were subjected to a standard superovulation regimen as described previously and the oocytes were collected from the ampulla of oviducts 14 h after human chorionic gonadotropin (hCG) injection (Li et al., 2020).

### Cloning and Expression of *Eml1* and *Nudc*

Mouse *Eml1* and *Nudc* ORF were amplified by PCR using the cDNA derived from the normal WT GV-stage oocytes, and cloned into the pCMV6-AC-3DDK and pCMV6-AN-mkate vector (Origene), respectively. Ectopic expression of the EML1-3DDK and NUDC-mKate fusion proteins in HEK293T cells was then achieved by transfection with 10 μg of the plasmid DNA using the Megatran1.0 (Origene) transfection reagent. Successful expression of the fusion protein was confirmed by either IF staining or WB analysis using the anti- FLAG (DDK) or tRFP (mKate) antibody.

### Microinjection of *Eml1*-MO and *Eml1*-3DDK mRNA

Morpholino oligomers for EML1 (EML1-MO, 5′-TAG CTGGAGAAGCCGTCCTCCATGC-3′) and the Standard Control (Control-MO, 5′-CCTCTTACCTCAGTTACAATTT ATA-3′) were purchased from Gene Tools, LLC, and were dissolved in sterile water to a final concentration of 2 mM. *Eml1-*3DDK mRNA was synthesized as described previously (Guo et al., 2018), and diluted to a final concentration of 500 ng/μl for microinjection. Approximately 10 pl of MOs or mRNA was microinjected into the cytoplasm of one normal WT- FGO that was plating in the M2 medium containing 10% FBS. After microinjection, the oocytes were first maintained at GV-stage by incubation in 5 μM milrinone-containing medium for 20 h and 12 h, respectively, in order to let the MOs and mRNA fully function. The oocytes were then released from the milrinone medium, and cultured in maturation medium for up to 14 h.

### Assessment of Oocyte Microtubule Dynamics and Kinetochore-Microtubule Attachments

To investigate whether the subcellular localization of EML1 is dependent on the stability of microtubules, M I oocytes were treated with reagents that interfere with microtubule stability at 37°C, i.e., 20 mg/ml of Nocodazole for 10-15 min and 10 mM of Taxol (Selleck, #S1150) for 45 min, respectively, and the localization of EML1 was then examined by IF analysis. MI oocytes that were treated with the same concentration of DMSO under the same culture conditions served as Controls. To assess kinetochore-microtubule attachment, M I oocytes were subjected to cold treatment on ice for about 10 min, and then immediately fixed in 4% PFA for 30 min at room temperature. These oocytes were subsequently stained by IF for kinetochores and microtubules as described previously (Li et al., 2020).

### Immunofluorescence Analysis

Oocytes were fixed in 4% PFA in PBS for 30 min at room temperature, or subjected to treatment in PHEM buffer (60 mM PIPES, 25 mM HEPES, 10 mM EGTA and 2 mM MgCl2, pH 6.9) containing 1% (v/v) Triton X-100 for 10 min before fixation, followed by permeabilization and blocking for 1 h in PBS containing 0.1% Triton X-100 and 10% fetal bovine serum (FBS). The samples were subsequently incubated with primary antibodies (4°C, overnight) and Alexa flour 594/488-conjugated secondary antibodies (room temperature, 1 h), respectively, according to experimental design, and counterstained with Hoechst 33342 for 10 min to label chromosomes. All images were taken under a LSM710 META confocal laser-scanning microscope (Zeiss) with the same settings. Data analysis was performed using ZEN 2.6 (blue edtion) LSM and ImageJ software (National Institutes of Health, United States) under the same processing parameters.

### Western Blot Analysis

Western Blot (WB) analysis was performed as described previously (Guo et al., 2018). Briefly, oocyte samples were lysed in 2 × Laemmli sample buffer, and heated at 108°C for 5 min to be denatured. The proteins were separated by SDS-PAGE and transferred onto polyvinylidene difluoride (PVDF) membranes for probing the proteins under examination. The expression of β-actin (ACTB) served as internal control of each sample. Quantification of the intensity of the protein band of interest detected by WB was accomplished using Image J software according to the instructions provided by the manufacturer.

### Co-immunoprecipitation and Mass Spectrometry

HEK293T cells transfected with *Eml1*-3DDK and *Nudc*-mKate plasmid DNAs were harvested and lysed using the lysis buffer that comes with the Pierce^TM^ Crosslink Immunoprecipitation Kit (Thermo Fisher Scientific, #26147). Immunoprecipitation (IP) was then carried out on these cell lysates using the anti-FLAG (DDK) M2 and anti-tRFP/mKate antibodies, respectively. The IP products were then subjected to WB validation followed by Mass Spectrometry analysis. For Mass Spectrometry analysis, the IP products from two independent experiments were resolved simultaneously on 10% SDS-PAGE and visualized with Coomassie blue. The lanes corresponding to each IP product were sliced out and sent to the proteomics core facility of the Institute of Biomedical Sciences at Fudan University (Shanghai, China) for Mass Spectrometry analysis.

Mass Spectrometry analysis was carried out using the same exact protocols as detailed in the previous studies (Zhang et al., 2007; Wu et al., 2018). Briefly, the gel slices were cut into 1 mm^3^ particles, destained, reduced, and alkylated, followed by the overnight Trypsin in-gel digestion at 37°C. LC-ESI-MS/MS analysis was performed using a nanoflow EASY-nLC 1000 system (Thermo Fisher Scientific, Odense, Denmark) coupled to an LTQ Orbitrap Elite mass spectrometer (Thermo Fisher Scientific Bremen, Germany). The raw data were analyzed by Proteome Discoverer (version 1.4, Thermo Fisher Scientific) using an in-house Mascot Server (version 2.3, Matrix Science, London, United Kingdom) (Perkins et al., 1999). Human database (20160213, 20,186 sequences) was downloaded from UniProt. Data were searched using the following parameters: trypsin/P as the enzyme; up to two missed cleavage sites were allowed; 10 ppm mass tolerance for MS and 0.05 Da for MS/MS fragment ions; propionamidation on cysteine as fixed modification; oxidation on methionine as variable modification. The incorporated Target Decoy PSM Validator in Proteome Discoverer and the Mascot expectation value was used to validate the search results and only the hits with FDR ≤ 0.01 and MASCOT expected value ≤ 0.05 were accepted for discussion.

### Statistical Analysis

Statistical analysis was performed using GraphPad Prism 7.0 software (GraphPad Software, Inc, United States). Student’s *t*-test was conducted to compare differences between two groups. *P* < 0.05 was defined to be significantly different. Data presented are presented as Mean ± SEM of at least three independent experiments.

## Results

### Forward Genetics Identified EML1 as an Essential Regulator of Oocyte Meiotic Progression and Female Fertility in Mice

**FIGURE 1:**
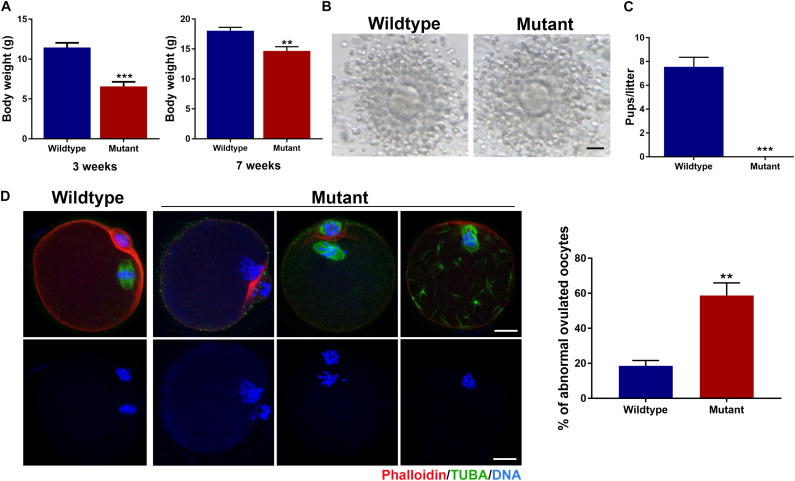
Growth and fertility defects in female mice carrying the ENU-induced nonsense mutation of *Eml1*. **(A)** Comparison of body weight between wildtype and *Eml1*-mutant (Mutant) female mice at the age of 3 and 7 weeks. **(B)** Microphotographs showing the ovulated cumulus-oocyte complexes by Wildtype and Mutant females. Scale bars indicate 10 μm. **(C)** Fecundity (pups/litter) of WT (*n* = 3) and Mutant (*n* = 3) female mice during the 2 month fertility test period. ***P < 0.005, compared with the Wildtype. **(D)** Assessment of spindle morphology and chromosome alignment in the Wildtype and Mutant ovulated oocytes. The left panel shows the representative micrographs of the IF stained oocytes. Spindles are stained in green, chromosomes and F-actins are stained in blue and red, respectively. Scale bars indicate 20 μm. The right bar graph shows the percentage of the oocytes with abnormal spindles and/or misaligned chromosomes. **P < 0.05, compared with the Wildtype.

The *Eml1*^tvrm360^ mutant mice were created by the ENU-induced mutagenesis in the Translational Vision Research Model (TVRM) program led by Dr. Patsy Nishina at The Jackson Laboratory. This ENU-induced mutant allele bears a nonsense point mutation (c.1956T > A; pTyr652^∗^) of *Eml1*, which creates a premature stop codon after pTyr652^∗^, and leads to the nonsense-mediated decay of *Eml1* mRNA (Collin et al., 2020). Therefore, these mutant mice present a nice model for addressing the role of EML1 in the regulation of oocyte meiotic progression and female fertility. Consistent with the reported low postnatal viability, we found that the *Eml1*^tvrm360^ mutant females are extremely small in size (6.55 ± 0.60g), with the body weight only about half of the wild type (WT) littermates (11.44 ± 0.59g, *P* < 0.01) at the age of 3 weeks. For those mutant females that survived to 7 weeks and beyond (14.68 ± 0.70g), the difference from the WT (18.04 ± 0.57g, *P* < 0.01) in body weight was significantly minimized (Figure 1A).

Using these precious 7-week and plus old mutant female mice, we analyzed the phenotypes of the oocytes and female fertility. We found that the *Eml1*^tvrm360^ mutant mice could ovulate following a standard superovulation regimen, *i.e.*, injection with 5IU/mouse eCG followed 5IU/mouse hCG within a 48-h interval. The ovulated cumulus-oocyte complexes (COC) appeared normal, with the well expanded cumulus oophori surrounding the mature oocytes (Figure 1B). Although most of the *Eml1*^tvrm360^ mutant mice died at about 4 months of age, fertility test by mating with normal B6D2F1 males indicated that the *Eml1*^tvrm360^ mutant females did not produce any pups or have any signs of pregnancy during the entire period of testing when they were still alive. In contrast, the female WT littermates of the *Eml1*^tvrm360^ mutants produced normally in the same period with an average of 7.5 pups/litter (Figure 1C). Whole mount immunofluorescene (IF) analysis of the spindle morphology and chromosome configuration indicated that near 60% (57.8 ± 7.25%, *P* < 0.01) of the oocytes ovulated by *Eml1*^tvrm360^ mutant females was abnormal. They displayed various abnormalities including partial or complete depolymerization of the microtubules and incomplete cytokinesis coupled with chromosome misalignment or failure of segregation (Figure 1D).

### Stable Expression and Subcellular Colocalization of EML1 With Meiotic Spindles in Oocytes During Meiotic Division

**FIGURE 2:**
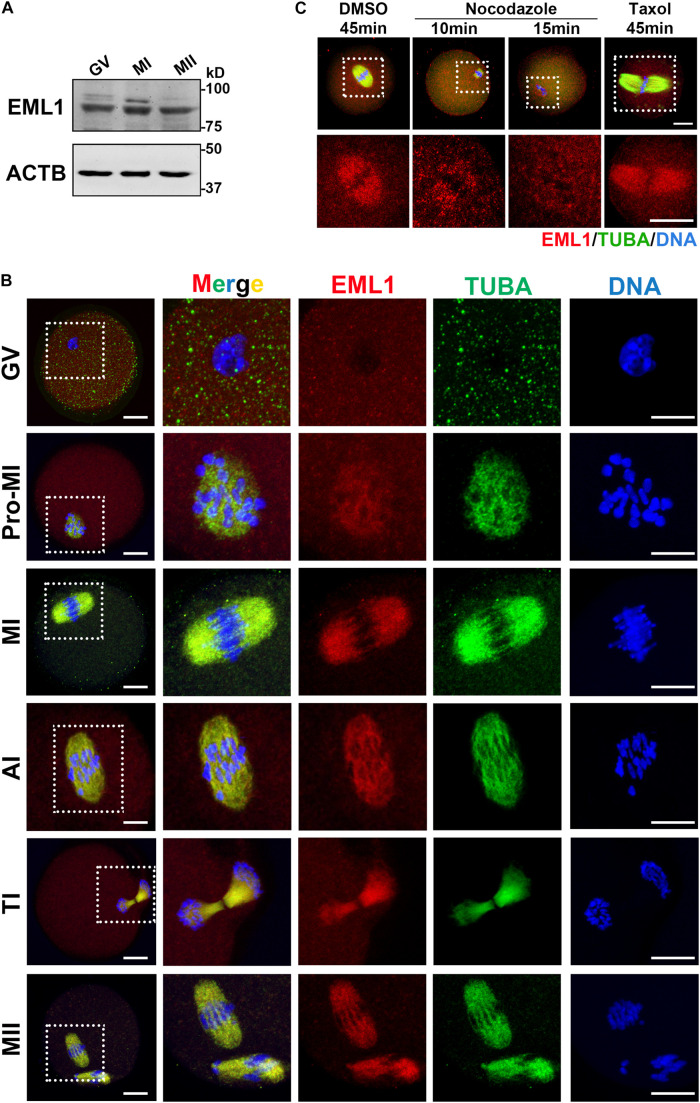
Stable expression and specific localization of EML1 in mouse oocyte during meiotic progression. **(A)** Western Blot (WB) analysis of the expression of EML1 and ACTB (internal control) protein in oocytes at different stages of maturation. Lysate of 200 oocytes that were collected at 0, 8, and 14 h of IVM corresponding to GV-, MI-, and MII- stage, respectively, was load in each lane. **(B)** Confocal micrographs showing the dynamic co-localization of EML1 protein with meiotic spindles in oocytes at various stages (i.e., GV, Pro-MI, MI, AI, TI, and MII) of maturation. EML1 and α-tubulin were stained in red and green, respectively, while chromosomes were stained in blue. The far left panel shows the whole-oocyte view of the staining. The magnified view of the boxed area within the oocyte at each stage is listed on the right side. Scale Bar = 20 μm. **(C)** Confocal micrographs demonstrating the changes of localization of EML1 and tubulin in MI- stage oocytes treated with microtubule-interfering drugs. MI- oocytes were treated with 20 μg/ml nocodazole for 10 and 15 min, respectively, or with 10 μM Taxol for 45 min, and then processed for IF analysis of EML1 and α-tubulin localization. EML1, α-tubulin, and chromosomes are shown in red, green, and blue, respectively. The first row indicates the whole-oocyte view of the staining. The magnified view of the boxed area within the oocyte is shown in the second row. Scale Bar = 20 μm.

The spindle and chromosome defects observed in the *Eml1*^tvrm360^ mutant oocytes suggest that EML1 may have specific subcellular localization during the process of meiotic division. We therefore examined the expression and subcellular localization of EML1 protein in mouse oocytes. As shown in Figure 2A, western blotting (WB) analysis showed that EML1 protein is expressed in the fully grown oocytes (FGO), and is maintained at stable levels during the process of meiotic progression. No apparent changes were observed among the immature germinal vesicle (GV), maturing metaphase I (MI), and matured MII stages oocytes. To investigate the subcellular distribution of EML1, whole mount IF was carried out on oocytes at various stages of meiotic division (Figure 2B). In the immature GV-stage oocyte, EML1 was ubiquitously distributed in the cytoplasm with no specific localization was observed. After the resumption of meiosis, EML1 was found to be colocalized with the nucleated microtubules, and enriched exclusively on the meiotic spindles at MI, anaphase/telophase I (AI/TI), and MII stages.

To test whether the specialized localization of EML1 within the maturing oocytes is dependent upon the integrity of the meiotic spindles, oocytes at MI stage were treated with either the microtubule depolymerization reagent, nocodazole, or microtubule stabilizer, Taxol, and the localization of EML1 was then examined. As indicated by Figure 2C, under the treatment with Nocodazole, oocyte spindles were shrinked and disappeared gradually, so did the specific localization of EML1 on the spindles. In contrast, taxol treatment stabilized the spindles, and accordingly, EML1 was persistently stayed on the enlarged meiotic spindles.

In order to verify whether the staining patterns by the EML1 antibody in oocytes were specific to EML1 protein, we microinjected the mRNA encoding C-terminal tagged EML1-3DDK fusion protein into oocytes and examined its expression and localization using the DDK antibody. Prior to this experiment, we first validated that *Eml1-*3DDK was correctly cloned and expressed using the HEK293 cell line. As shown in Supplementary Figure 1, WB analysis of the lysate from cells transfected with *Eml1*-3DDK plasmid DNA detected the expression of the fusion protein; while IF staining with the DDK antibody indicated that the fusion protein was localized on the mitotic spindles. In the oocytes that were injected with the *Eml1-*3DDK mRNA, IF staining with DDK antibody revealed the same specific localization pattern of EML1-3DDK with that directly detected by the EML1 antibody (Supplementary Figure 2).

### Knockdown of *EML1* in Oocytes Causes the Delay of Oocyte Resumption and Completion of the First Meiosis

**FIGURE 3:**
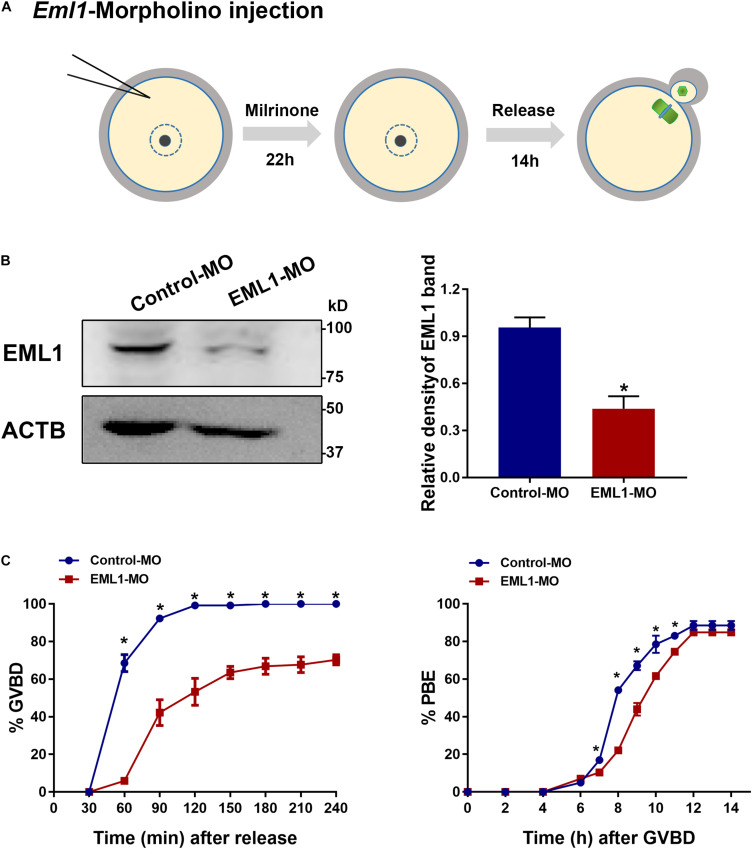
Knockdown of EML1 expression in oocytes causes the delay of meiotic progression. **(A)** Schematic illustration of the experimental design for examining the effect of EML1 knockdown on oocyte meiotic maturation. **(B)** WB analysis of the efficiency of knocking down EML1 in oocytes by EML1-morpholino oligos (MO). Lysate from 200 oocytes was loaded in each lane. ACTB serves as internal control. Representative gel image is shown in the left panel, whereas quantification of the WB result is shown in the right panel. *P < 0.05, compared with the Control-MO. **(C)** Effect of knockdown of EML1 in oocytes on the kinetics of GVB (left graph) and PBE (right graph). *P < 0.05, compared with the EML1-MO groups.

Because the severe rarity of obtaining sufficient number of viable *Eml1*^tvrm360^ mutant mice for experimentation, we turned to use the *in vitro* knockdown approaches to investigate the role of EML1 in the regulation of oocyte meiotic maturation. We knocked down the expression of EML1 in FGOs of normal WT females with the specific ATG-morpholino oligos (MO) that block translation (see Figure 3A for the illustration of the experimental design). WB analysis indicated that 22 h after microinjection, the levels of EML1 protein in the oocytes receiving EML1-MO were reduced by 57.1 ± 0.44%, which was significantly lower than the control group (*P* < 0.05; Figure 3B). When these injected oocytes were released from milrinone medium to undergo *in vitro* maturation (IVM), there was a significant delay in the kinetics of both the resumption and completion of the first meiosis, as indicated by germinal vesicle breakdown (GVBD) and extrusion of the first polar body (PBE) (Figure 3C). GVBD took place between 30-60 min after the initiation of IVM, and completed by 90-120 min (99.17 ± 0.83%) in the control group that receiving control morpholino (Conrol-MO). However, in the EML1-MO group, GVB wouldn’t occur until 60-90 min after IVM, and at all the time points been examined, the rate of GVB was much lower than that of the control groups. Moreover, for those oocytes that have already undergone GVB, PBE was also delayed in the EML1-MO group, with the rate at 7-11 h after GVBD being significantly lower than that in the controls.

### EML1 Depletion Impairs Spindle Assembly and Chromosome Alignment in MI Oocytes

**FIGURE 4:**
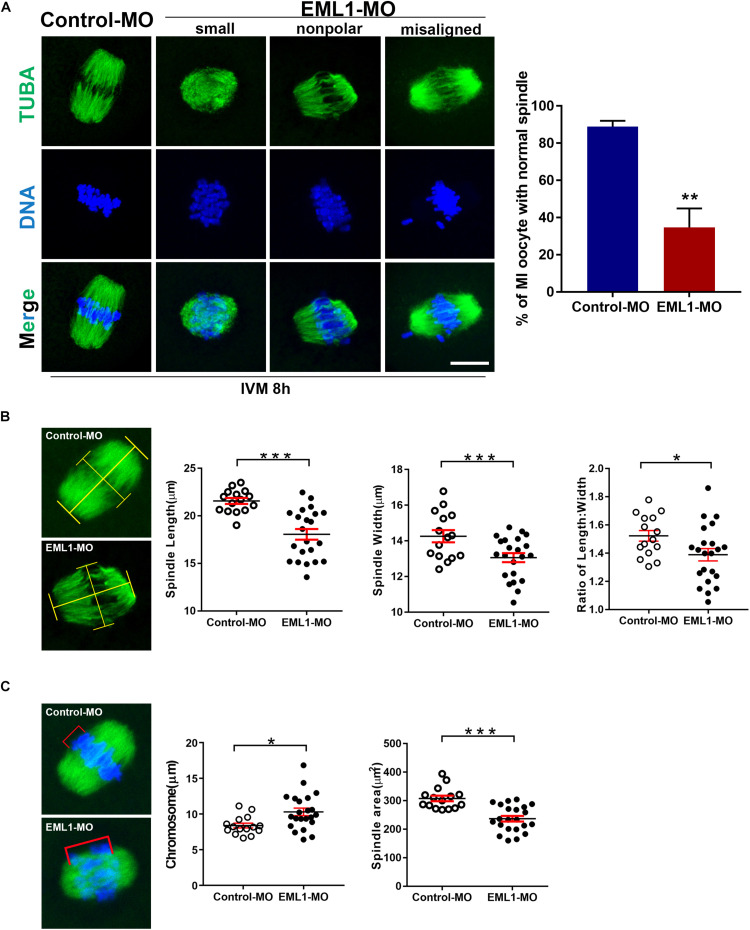
Knockdown of EML1 in oocytes impairs MI- spindle assembly and chromosome alignment. **(A)** Oocytes microinjected with the MOs were first maintained at GV-stage in milrinone-containing for 24 h, and then transferred to milrinone-free medium for IVM. After 8-h IVM, the oocytes were subjected to IF staining of spindles and chromosomes. The left panel is the representative micrographs of the IF staining, with the α-tubulin and chromosomes stained in green and blue, respectively. Arrows point to misaligned chromosomes. Scale Bar = 20 μm. The right bar graph shows the quantification of the percentage of the oocyte with normal spindles as revealed by the IF analysis. **P < 0.01, compared with the Control-MO group. **(B)** Geometric analysis of the MI- spindles in oocytes that were treated with Control-MO and EML1-MO and EML1-MO. Spindle (stained in green) length and width were measured as illustrated in the left micrographs, and plotted into dot graphs as shown in the right panels. *P < 0.05, ***P < 0.005. **(C)** Geometric analysis of chromosome alignment in MI- oocytes that were treated with Control-MO and EML1-MO. Chromosome displacement was measured according to the illustration in the left panel, and the quantification was shown in the right graph. The spindle was shown in green and chromosomes in blue. *P < 0.05, ***P < 0.005.

The formation of bipolar spindles and correct congression of chromosomes at MI stage is critical for normal progression and completion of the first meiosis. The significant delay of oocyte PBE after EML1 knockdown prompted us to assess whether spindle assembly and chromosome alignment were impaired in these oocytes. IF staining of α-tubulins in oocytes that have undergone IVM for 8 h revealed that 88.93 ± 3.07% of the oocytes in the control group formed normal appearing MI spindles with the homologous chromosomes all nicely aligned at the equator. However, in the EML1-MO treated group, only 34.73 ± 10.16% of them had normal spindles with well aligned chromosomes. In the rest of the EML1-MO treated oocytes, the spindles were either stunted in size or deformed with the spindle pole area extremely flat and some of the misaligned chromosomes stretched-out far away from the spindle equator (Figure 4A). This spindle and chromosome defect was further characterized by careful measurements of the length and width of the spindles, as well as the distance between the bivalents that fall farest apart, defined as “chromosome displacement.” These geometric measurements demonstrated that both the length and width of the spindles were reduced, while the chromosome displacement was significantly increased, in the EML1-knockdown oocytes (Figures 4B,C).

### EML1 Knockdown Disrupted the Normal Assembly of Microtubule Organization Center (MTOC) Onto the Spindle Pole

**FIGURE 5:**
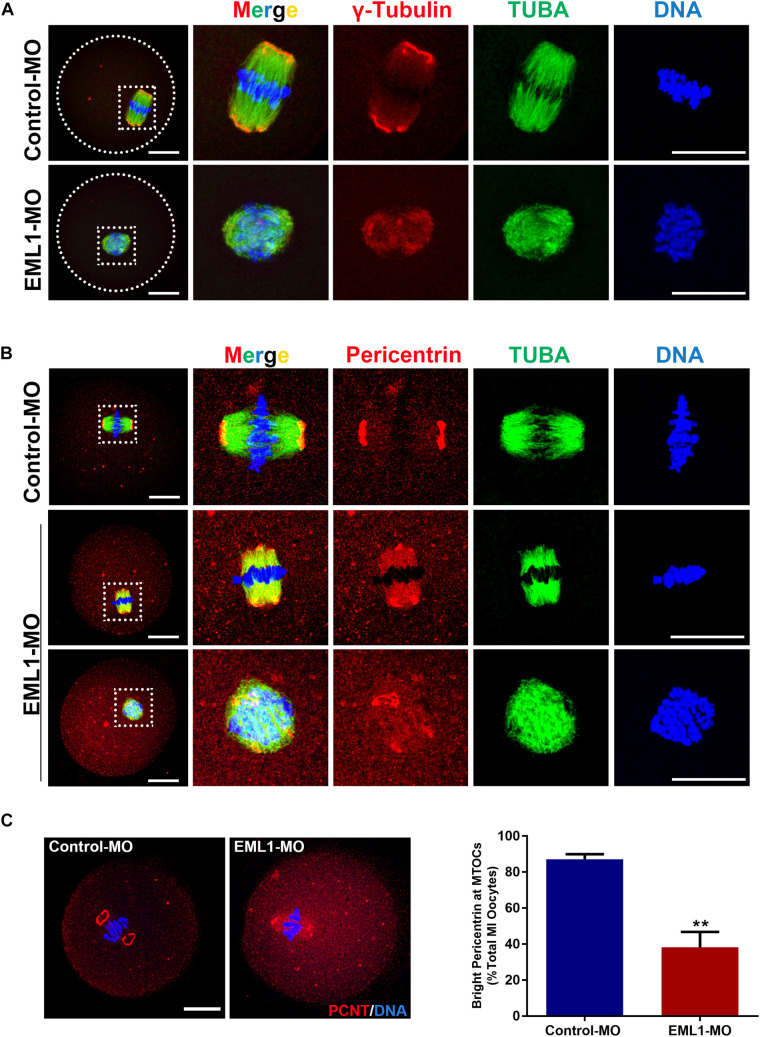
Knockdown of EML1 in oocytes disrupts the normal localization of a MTOC-associated proteins to the spindle pole. **(A)** Representative images of MO-injected oocytes labeled with anti-γ-Tubulin (red) and anti-α-Tubulin (green). DNA was counterstained with Hochest 33342 (blue). **(B)**. Representative images of MO-injected oocytes labeled with anti-Pericentrin (red) and anti-α-Tubulin (green). DNA was counterstained with Hochest 33342 (blue). In both **(A,B)**, the far left panel indicate the whole-oocyte view of the staining. The magnified images of the boxed areas within the oocyte are listed on the right side. Scale Bar = 20 μm. **(C)** Quantification of the percentage of oocytes with correct spindle pole Pericentrin (PCNT). **P < 0.01, compared with the Control-MO group.

Since the most typical phenotypes of EML1-delepted MI oocytes were small spindle and aberrant spindle pole, we speculated that the absence of EML1 may interrupt the normal function of MTOC in oocytes during meiotic progression. We hence examined the effect of EML1 knockdown on the intracellular distribution of the key MTOC components, *i.e.*, γ-tubulin and pericentrin, in oocytes. Consistent with previous reports by others, IF analysis showed that γ-tubulin was positively stained on the MI-spindle in the control oocytes, with prominent punctate foci concentrating at the spindle poles; while the staining pattern of pericentrin was more unique, with crescent- or horseshoe-shaped discrete foci detected exclusively at the spindle pole region. After the oocytes were treated with EML1-MO, the discrete foci of γ-tubulin staining at the spindle pole area was lost, and a more diffused distribution on the entire spindle area was formed (Figure 5A). The spindle pole area restricted pattern of localization for pericentrin was also disrupted by EML1-knockdown, with the positive foci either scattered around the pole area or diffused onto the body of the spindle (Figure 5B). This happened in 61.9 ± 8.60% of the EML1-MO treated MI-stage oocytes (Figure 5C).

### EML1 Depletion Interfered With the Attachment of Kinetochore to Microtubules and the Checkpoint of Spindle Assembly

**FIGURE 6:**
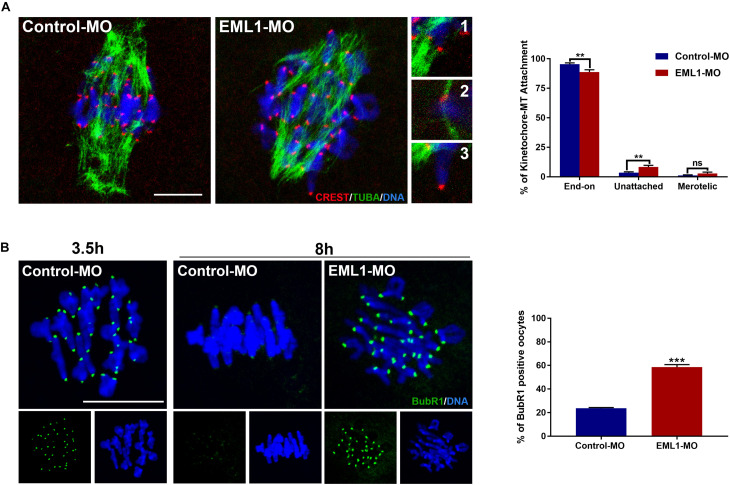
Increased kinetochore-microtubule attachment error and abnormal activation of spindle assembly checkpoint in EML1-Knocked down MI-stage oocytes. **(A)** Assessment of kinetochore–microtubule attachments in MO-injected oocytes that were matured *in vitro* for 8 h by IF staining. Microtubules, kinetochores, and chromosomes were stained in green, red, and blue, respectively. Representative images of end-on (1), unattached (2), and merotelic (3) attachments are shown. A total of 349 and 247 attachments were assessed, respectively, in the Control-MO and EML1-MO treated oocytes. **P < 0.01, ns denotes no significant difference. compared with the Control-MO and EML1-MO group. **(B)** Assessment of the activation of SAC in MO-injected oocytes that were matured *in vitro* for 8 h by IF staining of BubR1. Oocytes that were normally matured *in vitro* for 3.5 h served as positive control. BubR1 and chromosomes are stained in green and blue, respectively. ***P < 0.005, compared with the EML1-MO group.

The preceding observations of the delayed PBE and defects in spindle assembly and chromosome alignment in EML1-knocked down oocytes indicates that the kinetochore-microtubule (K-M) attachment and the spindle assembly checkpoint are probably also compromised in these oocytes. Indeed, we observed that the rate of kinetochores forming stable “end-on” type of attachment with spindle microtubules was reduced (Control-MO 95.36 ± 1.15% vs. EML1-MO 88.82 ± 1.92%, *P* < 0.01), while that having no microtubule attached was increased in EML1-MO treated oocytes (Control-MO 3.47 ± 0.76% vs. EML1-MO 8.27 ± 1.41%, *P* < 0.01) after cold treatment (Figure 6A). Also, IF staining of BubR1 on kinetochore revealed the aberrant inactivation of SAC in MI-stage EML1-knockdown oocytes. There were more oocytes stained positively by BubR1 antibody at the kinetochore region following EML1-MO treatment (Control-MO 23.62 ± 0.69% vs. EML1-MO 58.63 ± 2.04%, *P* < 0.01; Figure 6B).

### EML1-Deficiency Caused Spontaneous Formation of Pronucleus in Matured Oocytes After the First Meiotic Division

**FIGURE 7:**
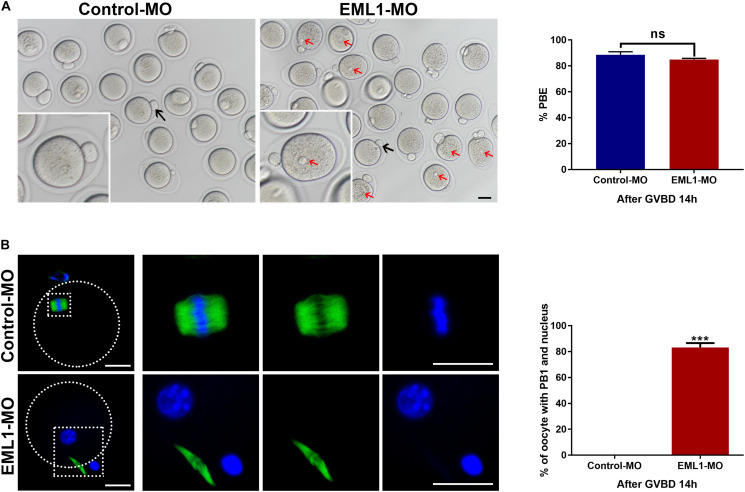
Knockdown of EML1 causes the spontaneous formation of pronucleus and failure to enter into the second metaphase by the matured oocytes after completion of the first meiotic division. Oocytes microinjected with Control-MO and EML1-MO were first maintained at GV stage for 24 h, and then transferred to maturation medium to allow for IVM. Oocytes that had undergone GVB within 2 h of IVM were selected and further cultured for additional 14 h. At the end of the culture, PBE and formation of pronucleus in oocytes were assessed. **(A)** Scoring PBE in cultured oocyte under light microscope. Representative bright field images of cultured oocytes are shown in the left panel, and the quantification of the rate of PBE are shown in the right bar graph. Red arrows denote pronucleus (PN), black arrows indicate PB1. Scale Bar = 10 μm. ns: non-significant. **(B)** Examining the formation of PN by fluorescent staining of tubulin (green) and DNA (blue) in the matured oocytes. The left panel is the representative images of the stained oocytes, with the magnified view of the circled area are listed on the right side. The right bar graph shows the quantification of the percentage of oocytes that have both the emitted PB1 and the PN. Scale Bar = 20 μm. ***P < 0.005, compared with the Control-MO group. ns denote no significant difference between the two groups compared.

Oocytes that were injected with EML1-MO extruded PB1 as normally as those receiving the Control-MO injection. However, almost all of the EML1 knocked down oocytes that have extruded PB1 contained a visible pronucleus (Figure 7A). IF staining revealed that 83.25 ± 3.34% of the EML1 knocked oocytes did not form the MII spindle after the first meiotic division, they instead entered interphase with the DNA completely de-condensed and the resulting secondary oocyte and the PB1 still connected by the central spindle (Figure 7B).

### Depletion of EML1 Compromised the Expression of CCNB1 and Phosphorylation of CDK1 in Oocytes

**FIGURE 8:**
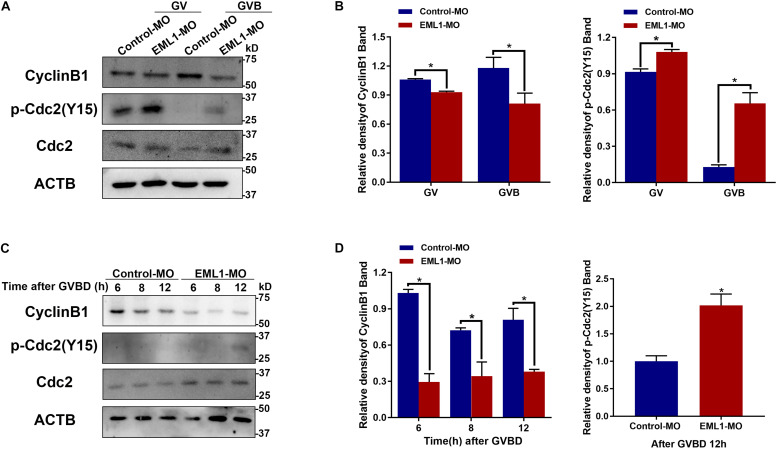
Depletion of EML1 in oocytes impairs the activation of MPF. Dynamic changes in the levels of CCNB1, pCDK1-Y15, and CDK1 in the oocytes during the period of oocytes meiosis resumption **(A,B)** and MI-to-MII transition **(C,D)** were assessed by Western Blot analysis, with the level of ACTB used as internal control. Lysate from 50 oocytes was loaded in each lane. The representative gel images of three independent experiments are shown in panels **(A,C)**, and the quantification of the Western Blot results are shown in panels **(B,D)**. *P < 0.05, compared with the Control-MO group.

Given the indispensable role of MPF in the control of oocyte meiotic resumption and progression, we tested the possibility whether depletion of EML1 affects the activation of MPF. Because MPF activity is determined by the steady-state levels of its regulatory subunit CCNB1 and the phosphorylation status of its catalytic subunit CDK1, we measured the levels of CCNB1 and the phosphorylated form of CDK1 by Western blot analysis. As shown in Figures 8A,B, after the oocytes were microinjected with EML1-MO and further cultured in milrinone-supplemented medium for 24 h, the levels of CCNB1 were slightly reduced (12.15 ± 0.08%, *P* < 0.05), while those of pY15-CDK1, the inactive form of CDK1, were increased (18.31 ± 0.09%, *P* < 0.05). When the oocytes were released from the milrinone medium and subjected for *in vitro* maturation, the differences between the control-MO and EML1-MO treated oocytes in the levels of CCNB1 (37.41 ± 0.15%, *P* < 0.05) and pY15-CDK1 (52.55 ± 0.09%, *P* < 0.05) became larger. Near complete dephosphorylation of pY15-CDK1 occurred concurrently with GVBD in the control oocytes, whereas in the EML1-MO group, this was significantly delayed. At the time (12 h after GVBD) when PB1 was normally extruded, pY15-CDK1 remained undetectable while CCNB1 stayed at a relatively high level in the control oocytes. However, in the EML1-MO treated oocytes, the levels of CCNB1 were still lower (0.8088 ± 0.01706 in the control vs 0.3803 ± 0.01874 in the EML1-MO, *P* < 0.05), while those of pY15-CDK1 were bounced back staying at a level higher than the control’s (2.018 ± 0.1465 fold of the control group, *P* < 0.05) (Figures 8C,D).

### Inhibition of WEE1/2 Kinases Partially Rescued the Defects of Meiotic Progression in EML1-MO Treated Oocytes

**FIGURE 9:**
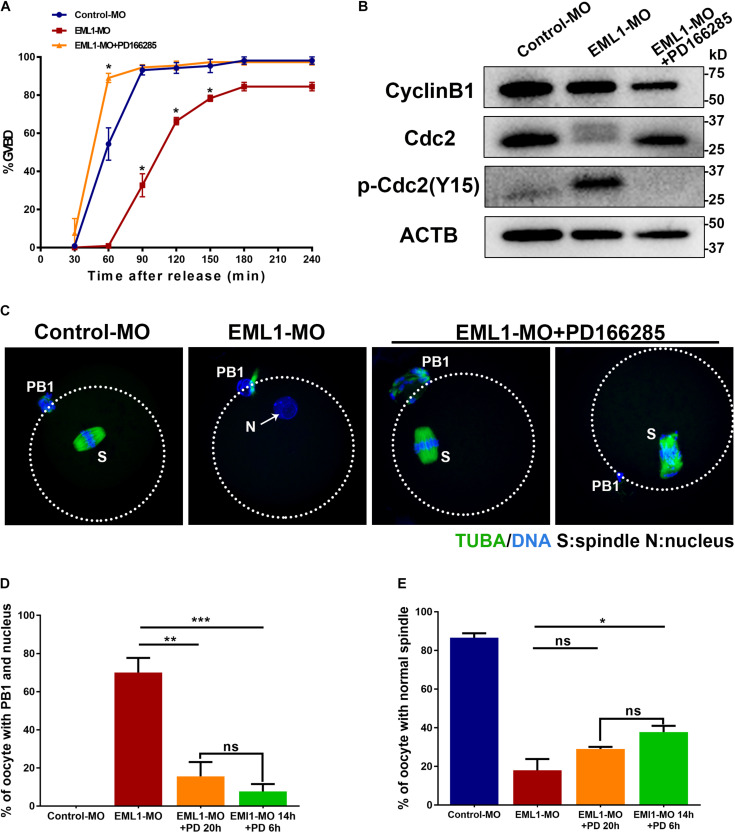
Activation of MPF by inhibitors of WEE1/2 kinases rescues the defects of meiotic progression in EML1-knocked down oocytes. **(A)** Oocytes microinjected with Control-MO and EML1-MO were first maintained at GV stage for 20 h, and then transferred to maturation medium with (for EML1-MO group) or without (for Control-MO group) the supplementation of the inhibitor of WEE1/2 kinases, PD166285, to allow for maturation. Oocyte GVB was scored during the culture. *P < 0.05, compared with the Control-MO group. **(B)** Western Blot analysis of the changes in the levels of CCNB1, pCDK1-Y15, and CDK1 in the EML1-knocked down oocytes that were treated with PD166285. ACTB was used as the loading control. Lysate from 50 oocytes was loaded in each lane. The representative gel images of three independent experiments are shown. (**C–E)** Assessment of the effect of PD166285 on the progression of meiosis to MII in EML1-knocked down oocytes. Oocytes microinjected with Control-MO and EML1-MO were first maintained at GV stage for 24 h. Then the EML1-MO oocytes were split into two groups, one of which was transferred to maturation medium supplemented with PD16628S and directly cultured for 20 h, while the other one was first transferred to maturation medium without PD166285 for 14 h, and then in the PD166285-supplemented medium for another 4 h culture. PBE, PN formation, and spindle morphology were then scored and analyzed. Representative images of the oocytes with the microtubules labeled with anti-tubulin antibody (green) and DNA stained with Hochest (blue) are shown in panel **(C)**. Scale Bar = 20 μm. Quantification of the percentages of oocytes with PB1 and PN, and those with normal MII-spindles are shown in panels **(D,E)**, respectively. *P < 0.05, **P < 0.01, ***P < 0.005, compared with the EML1-MO group. ns denote no significant difference between the two groups compared.

Results of the preceding experiments suggest that lower levels of MPF activity is probably the major causes of the meiotic defects observed in EML1-MO treated oocytes. We therefore tested this possibility by treating the EML1-knockdown oocytes with PD166285, a small molecule compound proven to be a specific inhibitor of WEE1 and 2 kinases, and shown to be able to activate MPF in mouse oocytes (Adhikari et al., 2014; Hanna et al., 2019). The result showed that treatment with 10 μM PD166285 effectively prevented the delay of GVBD in EML1-MO treated oocytes (Figure 9A). More interestingly, PD166285 even promoted the resumption of meiosis in EML1-knocked down oocytes, with the rate of GVBD already reaching 89.02 ± 4.17% within 1 h of IVM, which is significantly higher than that in the controls. Western blot analysis revealed that PD166285 treatment did not affect the expression of CCNB1 and CDK1, but dramatically reduced the levels of pY15-CDK1 in EML1-knocked down oocytes at the end of 14 h- IVM (Figure 9B). Coincidently, treating the EML1-knocked down oocytes with PD166285 at the beginning of IVM when the oocytes were still at GV stage, or 14 h after IVM when the oocytes already formed PN, effectively drove meiosis in these oocytes to progress to MII by the end of 20 h culture (Figures 9C,D). However, IF staining demonstrated that few of these PD166285 treated oocytes formed normal MII spindles with the chromosomes well aligned on the spindle equator (Figures 9C,E).

### Interaction and Co-localization of EML1 With NUDC

**FIGURE 10:**
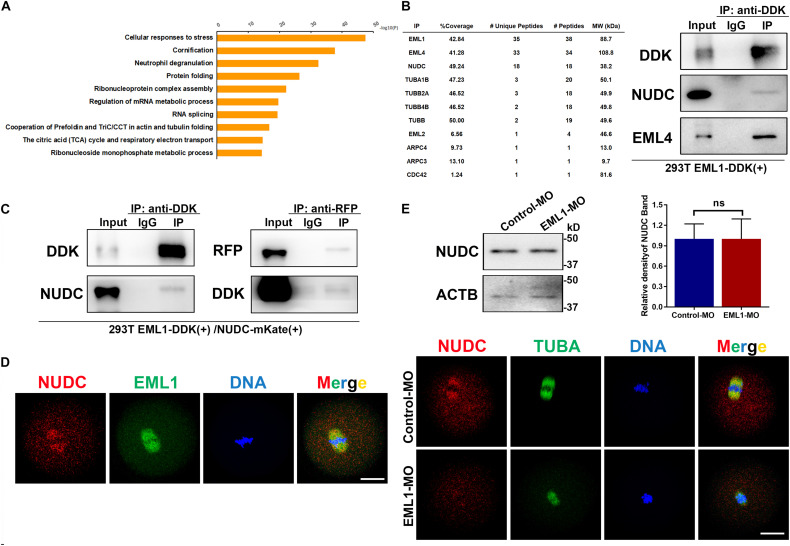
Potential interacting partners identified for EML1. **(A)** Gene enrichment analysis of the interacting proteins of EML1 identified by IP-MS. **(B)** List of IP-MS identified EML1interacting proteins that are involved in spindle morphogenesis and actin polymerization (left table), and the validation of the presence of NUDC and EML4 in the EML1-IP product by WB analysis (right panel). **(C)** WB validation of the interaction between EML1 and NUDC. Expression of 3DDK-tagged EML1 and mKate-tagged NUDC proteins was achieved by simultaneously transfecting HEK293T cells with the corresponding plasmids. Co-IP and WB analysis were then carried out with the anti-DDK and anti-mKate antibodies. **(D)** IF staining images showing the co-localization of NUDC with EML1 on the spindles of MI-stage oocytes. **(E)** Knockdown of EML1 did not affect the expression levels of NUDC in the oocyte as indicated by the WB analysis in the top panel, but dislodged the localization of NUDC from the spindles in oocytes (bottom IF images). ns, denote no significant difference between the two groups compared. Scale Bar = 20 μm.

To unravel the molecular mechanism of EML1 function, immunoprecipitation (IP) of EML1 protein followed by mass spectrometry (MS) analysis was carried to identify the potential interacting partners of EML1 using HEK293 cells expressing 3DDK tagged EML1. This resulted in the identification of a total of 514 proteins that were presumably partners of EML1 (Table S1). Gene enrichment analysis revealed that these proteins are mainly involved in “processes of RNA or mRNA metabolism/splicing/localization,” “composition of cytoplasmic ribosomal subunit and CDC5L complex,” and “protein folding and translation” (Figure 10A). Further mining of the protein list revealed that in addition to the proteins commonly known to be involved in the regulation of cytoskeleton dynamics, *i.e.*, tubulin protein TUBA1, TUBB, TUBB2, and TUBB4, actin polymerizion regulator ARPC3, ARPC4, and CDC42, as well as other members of the EML family (*i.e.*, EML2 and EML4), EML1 also interacts with NUDC (nuclear distribution C), an evolutionarily conserved *Nudc* gene product essential for cell division (Fu et al., 2016; Figure 10B). NUDC was recently reported to be highly expressed in mouse oocytes, and was suggested to be involved in the regulation of oocyte development (Cao et al., 2020). We therefore validated the interaction of EML1 with NUDC, and examined the potential co-localization of these two proteins in oocytes. The result of Co-IP experiment indicated that EML1 indeed interacts with NUDC (Figure 10C). IF staining showed that NUDC was co-localized with EML1on the spindles of MI-stage oocytes, and was dislodged from the spindles in EML1-knocked down oocytes (Figure 10E). Nevertheless, the expression of NUDC was not changed by EML1-knockdown in oocytes (Figure 10D).

## Discussion

Despite of the initial finding of the founding member of EMLs in the eggs and early embryos of the echinoderm species, the functional roles of the EML family proteins in the oocyte were heretofore largely undefined. We revealed here that EML1 is a bona fide MAP in mouse oocytes that is indispensable for the correct assembly of acentriolar bipolar spindles and the normal progression of the first meiosis to MII. These critical roles of EML1 seems to be mediated by different mechanism, with the former likely via interactions with other important factors for spindle morphogenesis and organization of the MTOC component at the spindle pole, whereas the latter possibly through a distinct mechanism involving the regulation of the activation of MPF.

Decoration of microtubules is a well-recognized feature common to the EML family proteins. Consistent with this notion, we observed in the present study that EML1 protein is steadily expressed and specifically localized on the spindles during the process of oocytes meiotic maturation in the mouse. The specificity of the spindle localization was demonstrated by IF staining of both the endogenous EML1 using the EML1 antibody and the ectopically expressed DDK-tagged EML1 using the DDK antibody in the oocytes. The association of EML1 with the spindle microtubules was also supported by the observation that EML1 changes its localization in the same way as the microtubules when the stability of oocyte spindles was interfered with Nocodazole and Taxol. Interestingly, we found that, unlike EML3, EML4 and sea urchin EMAP that were reported to be hyperphosphorylated in mitosis (Brisch et al., 1996; Pollmann et al., 2006), EML1 seems to be not phosphorylated during oocyte maturation since no apparent shift in its mobility was observed on the WB gel. This result suggests that the association of EML1 with meiotic spindles is probably not regulated by phosphorylation event postulated previously for this family of proteins. EML1 may hence have distinct roles in oocyte meiosis and function via different molecular mechanisms. Indeed, we observed that knockdown of EML1 in the fully grown oocytes *in vitro* causes the delay of both GVB and PBE, thus suggesting that part of the function of EML1 in oocytes is to ensure the precise timing of oocyte meiotic re-initiation and completion of the first meiotic division. More strikingly, we found that coincident with completion of the first meiotic division, EML1 knocked down oocytes fail to proceed to MII stage, but instead enter into the interphase and form PN. Chromosomes are decondensed in both the oocytes and the extruded first PBs. Therefore, EML1 is also essential for maintaining the dyads at condensed state after the segregation of the homologous bivalents, and preventing the secondary oocytes from entering into the interphase.

MPF is a cell cycle master regulator, its activation at late GV stage and reactivation at the end of first meiotic division are essential for oocyte meiotic resumption, and the transition into and arrest at MII, respectively (Adhikari and Liu, 2014; Adhikari et al., 2014). MPF activity is determined in large part by the levels of CCNB1 regulatory subunit and the phosphorylated forms of CDK1 catalytic subunit, with high levels of CCNB1 and low levels of p-CDK1 (Y14, Y15) beneficial to its activation (Adhikari and Liu, 2014). Our WB analysis of the CCNB1 and pCDK1-Y15 revealed that the levels of CCNB1 are significantly reduced in EML1 knocked down oocytes, especially after the resumption of meiosis. Also, a dramatic increase in the levels of pCDK1-Y15 is detected in the EML1 knocked down oocytes shortly after GVB, as well as at the time when oocytes normally reached the stage of MII. These changes in the levels of CCNB1 and pCDK1-Y15 in the EML1 knocked down oocytes are strong indicative of the reduction of MPF activity. It is therefore rational to believe that the presence of EML1 is required for maintaining the activity of MPF at optimal levels crucial for oocyte meiotic resumption and progression to MII. This inference is further buttressed by the observation that WEE1/2 kinases inhibitor, PD166285, effectively prevented the phosphorylation of CDK1 at Tyr15 residue and the formation of PN in the EML1 knocked down oocytes, and drove the oocytes entering to MII stage. WEE2 is the oocyte-specific isoform of WEE kinases, which acts as the direct upstream negative regulator of MPF through phosphorylation of CDK1 at the Thr14 and Tyr15 residues (Han et al., 2005). WEE2 plays an essential role in the maintenance of oocyte meiotic arrest before the LH surge and in the oocyte exit from the MII arrest upon fertilization (Han and Conti, 2006; Oh et al., 2010, 2011).

It is not clear how EML1 may regulate the activity of MPF in mouse oocytes. Since both the levels of CCNB1 and pCDK1-Y15 were significantly changed in the EML1 knocked down oocytes, EML1 may regulate the activation of MPF by interaction with the MPF subunits CCNB1 and CDK1, or the upstream regulators of CDK1. Interestingly, sea urchin EMAP was reported to interact with CDK1 in the unfertilized eggs (Brisch et al., 1996), which makes this postulated “interaction model” a more attractive candidate mechanism. There are several cases in the literature that also demonstrated the spontaneous formation of PN in the oocytes. These include oocytes from the LT/Sv and related mouse strains, the *Mos*-knockout, and the oocyte-specific *Ccnb1* and *Mastl* knockout (Colledge et al., 1994; Hashimoto et al., 1994; Eppig et al., 1996, 2000; Adhikari et al., 2014; Li et al., 2018). The formation of PN in the former two mouse models differs from the latter’s; it actually belongs to spontaneous parthenogenetic activation, *i.e.*, exit from MII arrest (Colledge et al., 1994; Hashimoto et al., 1994; Eppig et al., 1996, 2000). *Ccnb1* and *Mastl* knockout oocytes failed to mature to MII but rather enter into interphase and form PN, the same phenotype as what we have observed here (Adhikari et al., 2014; Li et al., 2018). Given that MASTL regulates the activity of MPF by antagonizing the activity of protein phosphatase PP2A, it is plausible to speculate that, in addition to CCNB1, EML1 may interact with MASTL to regulate the activity of MPF. Interestingly, EML1 was found by the pulldown assay in a very recent study to interact with MASTL in the embryonic brain extract (Bizzotto et al., 2017). This report adds more excitement to targeting MASTL as the potential interacting factor for EML1 to regulate MPF activity. Whether or not EML1 interact with MASTL, CCNB1, and CDK1 in mouse oocyte warrants further exploration.

Another important role that we revealed for EML1 is the regulation of the correct assembly of meiotic spindles in mouse oocytes, which is demonstrated by both the *in vivo* mutant model and the *in vitro* knockdown system. *In vitro*, knockdown of EML1 expression in the FGOs caused severe defects in spindle assembly at MI stage. The defects were reflected by small-sized spindles, flat spindle poles with disorganized localization of γ-tubulin and pericentrin, unattached kinetochores and misaligned chromosomes, and the abnormal activation of SAC. Therefore, EML1 is crucial for MI spindle assembly in oocytes. The sequentially connected defects in K-M attachment, chromosome alignment, and SAC activation may cumulate in the delay of anaphase I onset and the extrusion of the PB1 in the EML1 knocked down oocytes. Analysis of the oocytes ovulated by EML1-mutants revealed that EML1 is also essential for the formation and integrity maintenance of MII spindles. About 60% of the mutant ovulated oocytes are abnormal, with the chromosomes severely misaligned and the microtubules poorly organized. Because of the extreme scarcity of viable homozygous animals for experimentation, we were unable to use the *Eml1*-mutant mouse model to carry out detailed analysis of all stages of oocyte other than just focusing on the ovulated eggs. It is also important to note that phenotypic variations exist between the *Eml1*-mutant mouse oocytes and the *in vitro* EML1-knockdown oocytes. The discrepancy in the phenotype could be caused by the fundamental difference in the two model systems used. The *in vivo* mutant mouse model could reflect the systemic effect of *Eml1* mutation on oocyte throughout the entire process of oocyte and follicle development. While the *in vitro* EML1-knockdown in the normal fully grown oocytes could only reveal the acute effect of loss of EML1 during the specific process of oocyte development, *i.e.*, meiotic maturation. Given that all six members of the EML family proteins are expressed in the oocytes and/or granulosa cells, some functions of EML1 that are critical for oocyte meiotic progression to MII could be compensated by the other EML family members during the growth phase of the mutant oocytes. This compensation could probably happen at the levels of transcription in the growing oocytes *in vivo*, which could not be done by the fully grown oocytes *in vitro* upon EML1 is knocked down since these oocytes are transcriptional quiescent. Nevertheless, the mutant model complement well with the *in vitro* knockdown experiment in which the oocytes are unable to progress to MII. Further dissection of the physiological role of EML1 in the entire process of oocyte meiotic progression necessitates the creation of a female germ cell-specific knockout allele of *Eml1* in the future studies.

In a spontaneous mouse mutant model where the expression of *Eml1* was disrupted by a transposon insertion, the localization of γ-tubulin and the length of metaphase spindles were also found to be altered in the apical progenitors of the developing cerebral cortex (Kielar et al., 2014; Collins et al., 2019). But the spindles in this mutant brain cells were abnormally long, which was opposite to what we observed here in the oocytes with EML1 knocked down. This discrepancy might be due to the lack of centrioles in the oocytes since the astral microtubules nucleated at the centrosomes in the mitotic somatic cells can interact with the cell cortex and orient the mitotic spindles (Szollosi et al., 1972; Gonczy, 2002; Roubinet and Cabernard, 2014). Despite of this difference, it is clear that EML1 participates in the regulation of microtubule dynamics and spindle assembly in both mitosis and meiosis. The extremely flat spindle poles and the increased incidence of unattached kinetochores observed in the EML1 knocked down oocytes indicate that EML1 might be required for the nucleation and assembly of both the minus- and plus- end microtubules of the spindle. Knockdown of EML1 also disrupted the correct aggregation of γ-tubulin and pericentrin, two key components of the aMTOC in mouse oocytes. Given the indispensable roles of γ-tubulin and pericentrin in the control of microtubule nucleation and the recruitment of other key pericentriolar materials (*e.g.*, γ-tubulin, NEDD and CEP125) to the aMTOC, respectively, in mouse oocytes (Ma et al., 2010; Ma and Viveiros, 2014; Baumann et al., 2017), it is tempting to speculate that EML1 may regulate the assembly of oocyte acentriolar spindles by cooperation with γ-tubulin and/or pericentrin.

We identified here through IP-MS and validated by Co-IP analysis that EML1 interacts with NUDC, an evolutionally conserved gene product that is phosphorylated by PLK1 and Aurora kinase B, and is critical for K-M attachment, chromosome congression, spindle organization and cytokinesis during mitosis in mammalian somatic cells (Aumais et al., 2003; Nishino et al., 2006; Weiderhold et al., 2016). We found that, in the oocytes, EML1 and NUDC were colocalized to the spindle in MI oocytes, and knockdown of EML1 disrupted the spindle-specific localization of NUDC without affecting its expression levels. This result suggests that the function of EML1 in the regulation of oocyte meiotic spindle assembly is probably mediated, at least in part, by interaction with NUDC. Similar results were also found for EML4 in Hela cells, where the association of EML4 with NUDC is required for the localization of NUDC to the mitotic spindle (Chen et al., 2015). Interestingly, our IP-MS analysis in this study also revealed that EML1 binds to EML4 and EML2. Furthermore, NUDC was recently reported to be highly expressed in mouse oocytes, and was suggested to be involved in the regulation of oocyte development (Cao et al., 2020). These data together make it more likely that EML1 cooperates with NUDC to regulate oocyte meiotic spindle assembly.

Although MAPs were initially discovered as proteins that bind to and stabilize microtubules, they are now believed to exert a large variety of functions through interaction with a plethora of proteins (Bodakuntla et al., 2019). This may exactly be the case for EML1 since its N-terminal TAPE (Tandem atypical propeller in EML) domain contains both the unique HELP (hydrophobic EML protein) motif and several WD40 repeats (Richards et al., 2014). The HELP motif binds to tubulins, while the WD40 repeats are presumed to mediate protein-protein interactions for many biological functions (Stirnimann et al., 2010). In addition to its direct regulation of microtubule dynamics, EML1 may act as a scaffold to recruit other regulators essential for spindle assembly and oocyte meiotic progression. The identity and function of the factors that interact with EML1, as well as the ways through which they cooperate with each other, await further studies. This study therefore provides new insights into the understanding of how MAPs regulate spindle morphogenesis and meiotic progression in mammalian oocytes.

## Data Availability Statement

The datasets presented in this study can be found in online repositories. The names of the repository/repositories and accession number(s) can be found below: ProteomeXchange PXD025159.

## Ethics Statement

The animal study was reviewed and approved by the Ethical Committee of Laboratory Animals and Animal Care and Use Committee of the Nanjing Medical University (NJMU).

## Author Contributions

Y-QS and HY conceived the study. HY, TZ, HW, XiH, XuH, XF, YY, HL, and LS performed the research. HY and Y-QS analyzed the data and wrote the manuscript. All authors contributed to the article and approved the submitted version.

## Conflict of Interest

The authors declare that the research was conducted in the absence of any commercial or financial relationships that could be construed as a potential conflict of interest.

## Supplementary Material

Supplementary Figure 1 Expression and localization of C-terminal tagged EML1-3DDK fusion protein in HEK293T cell line during mitosis. **(A)** Western Blot (WB) analysis of the expression of EML1, FLAG and ACTB (internal control) protein in lysate from HEK293T cells transfected with *Eml1*-3DDK plasmid DNA for 24 h. **(B)** Confocal micrographs showing the dynamic co-localization of EML1-3DDK fusion protein with spindles in HET293T cells during mitosis. FLAG and α-tubulin were stained in Red and green, respectively, while chromosomes were stained in blue. Scale Bar = 10 μm.

Supplementary Figure 2 C-terminal tagged EML1-3DDK fusion protein showed the same special localization in mouse oocyte during meiosis progression with EML1 antibody. Oocytes microinjected with *Eml1*-3DDK mRNA were first maintained at GV stage for 12 h, and then transferred to maturation medium. Confocal micrographs showing the dynamic co-localization of EML1-3DDK protein with meiotic spindles in oocytes at various stages (i.e., GV, Pro-MI, MI, AI, TI, and MII) of maturation. FLAG and α-tubulin were stained in Red and green, respectively, while chromosomes were stained in blue. The far left panel shows the whole-oocyte view of the staining. The magnified view of the boxed area within the oocyte at each stage is listed on the right side. Scale Bar = 20 μm.
