# Supplementary material for: Echinoderm Microtubule Associated Protein Like 1 Is Indispensable for Oocyte Spindle Assembly and Meiotic Progression in Mice
Source: Front Cell Dev Biol. 2021 May 28;9:687522. doi: 10.3389/fcell.2021.687522 (PMC8194061; doi:10.3389/fcell.2021.687522)
Supplement: Supplementary Figure 1 — Expression and localization of C-terminal tagged EML1-3DDK fusion protein in HEK293T cell line during mitosis. (A) Western Blot (WB) analysis of the expression of EML1, FLAG and ACTB (internal control) protein in lysate from HEK293T cells transfected with Eml1-3DDK plasmid DNA for 24 h. (B) Confocal micrographs showing the dynamic co-localization of EML1-3DDK fusion protein with spindles in HET293T cells during mitosis. FLAG and α-tubulin were stained in Red and green, respectively, while chromosomes were stained in blue. Scale Bar = 10 μm. [file Data_Sheet_1.PDF]

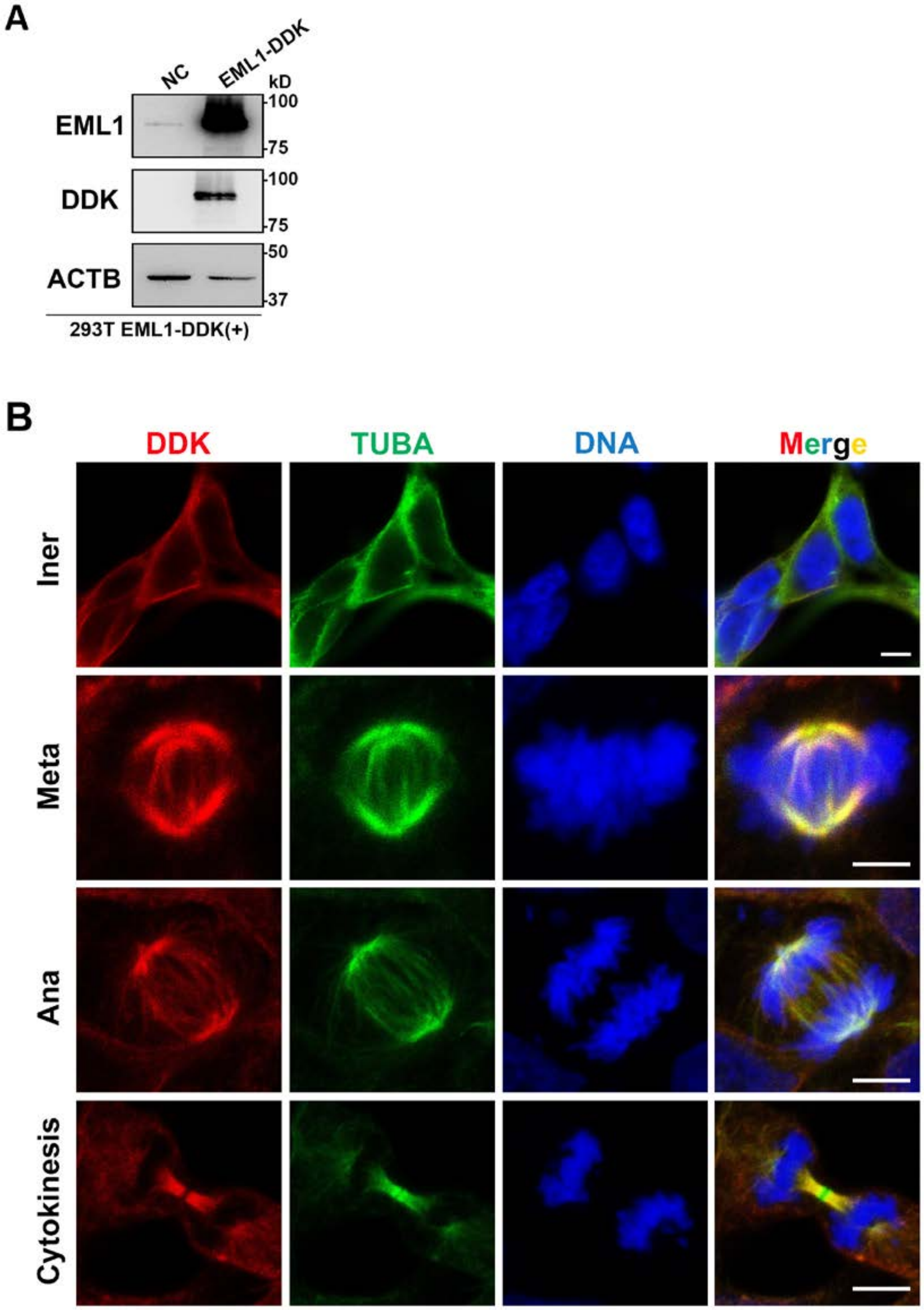

FIGURE S1. Expression and localization of C-terminal tagged EML1-3DDK fusion protein in HEK293T cell line during mitosis. **(A)** Western Blot (WB) analysis of the expression of EML1, FLAG and ACTB (internal control) protein in lysate from HEK293T cells transfected with *Eml1*-3DDK plasmid DNA for 24h. **(B)** Confocal micrographs showing the dynamic co-localization of EML1-3DDK fusion protein with spindles in HET293T cells during mitosis. FLAG and α-tubulin were stained in Red and green, respectively, while chromosomes were stained in blue. Scale Bar = 10 μm.

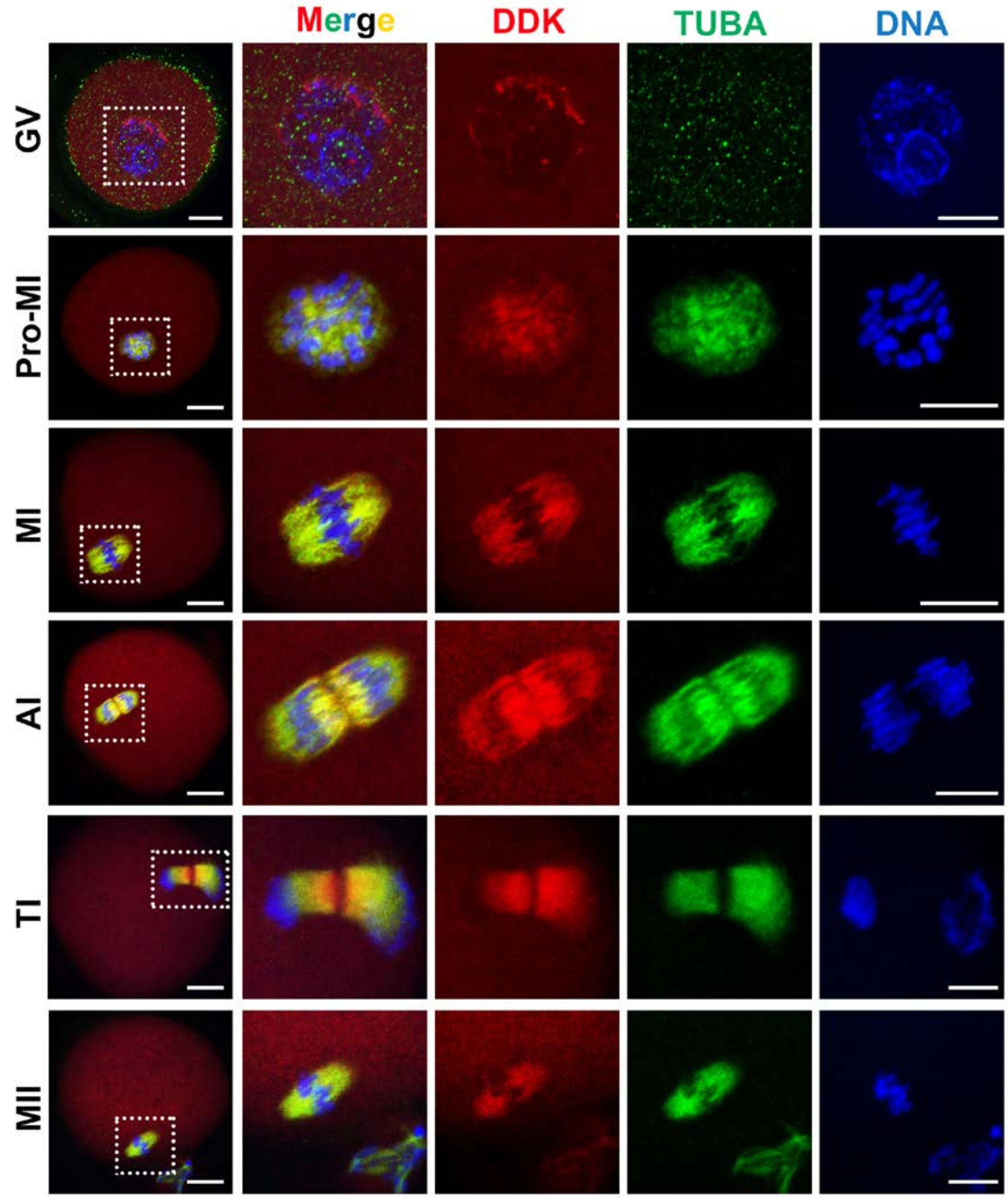

FIGURE S2. C-terminal tagged EML1-3DDK fusion protein showed the same special localization in mouse oocyte during meiosis progression with EML1 antibody. Oocytes microinjected with *Eml1*-3DDK mRNA were first maintained at GV stage for 12h, and then transferred to maturation medium. Confocal micrographs showing the dynamic co-localization of EML1-3DDK protein with meiotic spindles in oocytes at various stages (i.e., GV, Pro-MI, MI, AI, TI, and MII) of maturation. FLAG and α-tubulin were stained in Red and green, respectively, while chromosomes were stained in blue. The far left panel shows the whole-oocyte view of the staining. The magnified view of the boxed area within the oocyte at each stage is listed on the right side. Scale Bar = 20 μm.

This is a provisional file, not the final typeset article
